# Myocardial fibrosis in Eisenmenger syndrome: a descriptive cohort study exploring associations of late gadolinium enhancement with clinical status and survival

**DOI:** 10.1186/1532-429X-16-32

**Published:** 2014-05-14

**Authors:** Craig S Broberg, Sanjay K Prasad, Chad Carr, Sonya V Babu-Narayan, Konstantinos Dimopoulos, Michael A Gatzoulis

**Affiliations:** 1 Adult Congenital Heart Program, Knight Cardiovascular Institute, Oregon Health & Science University, Portland, Oregon, USA; 2 NIHR Cardiovascular Biomedical Research Unit, Royal Brompton and Harefield NHS Foundation Trust, Imperial College, London, UK; 3 Adult Congenital Heart Disease Centre and National Centre for Pulmonary Hypertension, NIHR Cardiovascular BRU, and the National Heart & Lung Institute, Imperial College, London, UK; 4 UHN 62, Knight Cardiovascular Institute, 3181 SW Sam Jackson Park Road, Portland, OR 97221, USA

**Keywords:** Eisenmenger, Cyanosis, Pulmonary artery hypertension, Myocardial fibrosis, Cardiovascular magnetic resonance

## Abstract

**Background:**

A relationship between myocardial fibrosis and ventricular dysfunction has been demonstrated using late gadolinium enhancement (LGE) in the pressure-loaded right ventricle from congenital heart defects. In patients with Eisenmenger syndrome (ES), the presence of LGE has not been investigated. The aims of this study were to detect any myocardial fibrosis in ES and describe major clinical variables associated with the finding.

**Methods:**

From 45 subjects screened, 30 subjects (age 43 ± 13 years, 20 female) underwent prospective cardiovascular magnetic resonance with LGE to quantify biventricular volume and function as well as maximal and submaximal exercise during a single visit. Standard cine acquisitions were obtained for ventricular volume and function. Further imaging was performed after administration of 0.1 mmol/kg gadolinium contrast. Regions of LGE were evaluated qualitatively and quantitatively by manual contouring of identified areas, with total area expressed as a percentage of mass. Patients were followed prospectively (mean follow up 7.4 ± 0.4 years) and any deaths recorded. Patients with LGE findings were compared to those without.

**Results:**

LGE was present in 22/30 (73%) patients, specifically in RV myocardium (70%), RV trabeculae (60%), LV myocardium (33%) or LV papillary muscles (30%), though in small amounts (mean 1.4% of total ventricular mass, range 0.16 – 6.0%). Those with any LGE were not different in age, history of arrhythmia, desaturation, nor hemoglobin, nor ventricular size, mass, or function. Exercise capacity was low, but also not different between those with and without LGE. Similarly no significant associations were found with amount of fibrosis. There were five deaths among patients with LGE, versus two in patients without, but no difference in survival (log rank =0.03, P = 0.85).

**Conclusions:**

Myocardial fibrosis by LGE is common in ES, though not extensive. The presence and quantity of LGE did not correlate with ventricular size, function, degree of cyanosis, exercise capacity, or survival in this pilot study. More data are clearly required before recommendations for routine use of LGE in these patients can be made.

## Background

Eisenmenger syndrome (ES) is a multisystem disorder. In addition to other vulnerable organs, myocardial dysfunction can occur from long-standing pressure loading and cyanosis, and heart failure is a common cause of eventual death [1]. As such, the etiology of ventricular dysfunction and its bearing on survival are of interest, including the process of myocardial fibrosis, which can be detected using cardiovascular magnetic resonance (CMR) with late gadolinium enhancement (LGE) [2].

Through several studies, LGE has been demonstrated in various congenital heart disease subgroups including those with tetralogy of Fallot, transposition of the great arteries, and following a Fontan palliation [3-6]. Collectively, these studies find that LGE is associated with several clinical markers of cardiovascular dysfunction, including ventricular enlargement, systolic dysfunction, poorer NYHA class, arrhythmia, elevations of brain natriuretic peptide and lower exercise capacity. Fibrosis appears to be an important player in the process of heart failure in adult congenital heart disease (ACHD) [7]. More diffuse microscopic fibrosis has also been demonstrated in ES [8].

Therefore, as part of a large prospective cohort study of Eisenmenger patients, we sought to define the prevalence and extent of LGE in ES and explore meaningful associations with other clinical or functional parameters and survival. We hypothesized that LGE would be more prevalent in patients with more cyanosis and ventricular dysfunction, and that it would portend worse survival.

## Methods

### Patients

Consecutive adults with ES, defined as a known intracardiac or extracardiac shunt with increased pulmonary vascular resistance and reversed or bi-directional shunt resulting in hypoxemia at rest, were invited to participate. Subjects were excluded for acute decompensated heart failure, active hemoptysis, recent surgery, or non-elective hospitalization, but were invited to participate after resolution of the event. Subjects with contraindications to CMR were excluded, including those with impaired renal function (creatinine > 1.5 mg/dl) who were ineligible for gadolinium contrast. Other elements from this prospective descriptive study have been previously reported [9,10]. The study was performed at Royal Brompton Hospital according to accepted clinical standards, after institutional ethics approval. All subjects gave signed informed consent prior to participation. Patients with developmental delay were allowed to participate if they were able to comply with verbal commands and provide oral assent with written consent signed by a parent or guardian. Data were obtained during a single intake visit.

### Cardiovascular magnetic resonance

CMR was performed using a 1.5 Tesla scanner (Sonata, Siemens, Erlangen, Germany) with a phased array body coil. After standard initial localizers and long-axis acquisitions using steady-state free precession, short-axis cine images (7 mm with 3 mm spacing) were obtained from base to apex. Volumes of the right ventricle (RV) and left ventricle (LV) at end-diastole and end-systole, indexed to body surface area, were calculated by contouring the epi- and endocardial borders from short axis images. Contouring included trabeculae and papillary muscles as part of the myocardial mass, per our standard protocol [3]. For patients with a ventricular septal defect (VSD), the same phase was used to define end-systole for both ventricles, and delineation between the RV and LV chambers at the defect was done using a line in direct continuity with the septum. The septum was included in LV mass quantification but hypertrophied trabeculae along the RV side were quantified as part of the RV mass. Phase-velocity mapping was performed in the main pulmonary artery to measure pulmonary blood flow.

LGE imaging was obtained 5–20 minutes after 0.1 mmol/kg IV gadolinium-DTPA (Magnevist, Bayer Schering Pharma, Berlin, Germany) administration. Multiple short-axis slices from base to apex were acquired using a segmented fast low-angle shot inversion recovery sequence (TE 4.3 ms, TR <90% of RR interval, FA 20, slice thickness 8 mm, 2 mm gap) as employed in other projects [3,4]. Inversion time ranged from 300 – 420 ms, adjusted as needed for appropriate nulling of normal myocardium. Presaturation pulse masking of cerebrospinal fluid was employed. Long-axis acquisitions were also obtained using the same sequence, as well as repeated short-axis planes with altered direction of phase encoding to clarify artifacts.

Images were analyzed offline using CMR tools (CMR Tools, London, UK) with contrast/brightness settings adjusted for uniform dark signal from normal myocardium. Images were compared to cine images in the same plane to ensure that small regions of enhancement were not areas of pooled gadolinium between trabeculae. LGE was defined as an area of increased signal within myocardium equal or greater to blood pool signal. Confirmation was obtained by comparing images with altered phase/frequency-encoding direction. Location and two-dimensional area of each region of enhancement were recorded. Total area was multiplied by slice thickness to give volume of LGE, converted to mass expressed as a percentage of total myocardial mass, similar to methods employed previously [3]. Enhancement was categorized by location within RV myocardium, RV trabeculae, LV myocardium, or LV papillary muscle, each expressed as a percentage of patients studied, with 95% confidence limits. Enhancement only within the RV-LV junction, a common finding in hypertrophy, was also noted but considered separately as it may not represent myocardial pathology in pulmonary hypertension [11].

### Exercise testing

All participants completed a sub-maximal 6-minute walk test. Distance walked was recorded in meters [12]. Patients also performed maximal exercise testing on a treadmill with a modified Bruce protocol with respiratory mass spectrometer (Amis 2000, Innovision, Odense, Denmark) for measurement of oxygen consumption (VO_2_), carbon dioxide production (VCO_2_), anaerobic threshold, peak VO_2_, and ventilatory efficiency slope (Ve/VCO2).

### Survival

Patients received follow-up at Royal Brompton Hospital, where detailed records were available for each. All patients were accounted for at the time of final analysis. For deceased patients, date of death and cause of death were obtained. Otherwise, date of last known clinical follow-up was recorded. Other standardized data in follow-up were not obtained.

### Statistical analysis

Analysis was done using SPSS for Windows 11.0. Continuous variables were compared with student’s t-test, while categorical variables were compared by chi-square or Fisher’s exact test. Non-normal variables (brain natriuretic peptide) were compared using the Mann–Whitney U test. Univariate regression was done using Pearson’s correlation coefficient. Results were expressed as mean ± SD or median/interquartile range, with p < 0.05 considered statistically significant. Variables associated with survival were identified using Kaplan-Meier analysis, though low numbers of events limited more in-depth analysis of survival.

## Results

Of 61 Eisenmenger patients screened, 45 patients met all inclusion/exclusion criteria for MRI scanning. From these 30 were successfully studied with LGE. Clinical and laboratory details are provided (Table 1). Reasons for no LGE imaging were patient tolerance with extended scanning or breath holding (N = 5), limited available scanner time (N = 4), poor image quality, usually as a result of limited ability to breath-hold (N = 4), renal insufficiency (N = 1), and provider preference (N = 1). Those without LGE imaging were younger (33 ± 10 vs. 43 ± 13 years, p = 0.014) and had a faster heart rate during the scan (88 ± 11 vs. 74 ± 12, p = 0.001), possibly reflecting increased anxiety during scanning. There was no difference in oxygen saturation, exercise capacity, ventricular size or function, or survival among those in whom LGE was or was not performed. Of those completing the LGE portion, three had malposed great arteries (double outlet right ventricle in two, and D-transposition with a VSD in one, who had undergone a palliative Mustard procedure). Two had developmental delay.

**Table 1:** Clinical characteristics and comparisons

	**All patients**	**RV myocardial enhancement**	**No RV myocardial enhancement**	**P**
	**N = 30**	**N = 21**	**N = 9**	
Age at baseline (years)	43.4 ± 13	43.4 ± 11.4	43.3 ± 17.8	0.99
Oxygen saturation (%)	81 ± 7	81 ± 7	81 ± 6	0.78
Female	20 (67%)	14 (67%)	6 (67%)	0.99
History of arrhythmia	3 (10%)	2 (9%)	1 (11%)	0.60
Hemoglobin (g/dl)	20 ± 2.6	20.3 ± 2.8	19.3 ± 2.1	0.34
Creatinine (μmol/l)	86 ± 24.6	86 ± 24	88 ± 28	0.81
Urea (mmol/l)	6.1 ± 2.8	5.9 ± 2.3	6.3 ± 3.5	0.75
Brain natriuretic peptide (pmol/l)*	15/14	14/24	15/13	0.99
RV ejection fraction (%)	51 ± 14	51 ± 13	50 ± 18	0.69
LV ejection fraction (%)	57 ± 11	59 ± 10	51 ± 12	0.06
P_A_CO_2_ (kPa)	4.4 ± 0.7	4.5 ± 0.6	4.1 ± 0.8	0.35
P_A_O_2_ (kPa)	6.4 ± 0.9	6.4 ± 0.9	6.4 ± 0.9	0.93
Exercise Duration (min)	5.7 ± 2.4	5.2 ± 2.1	6.2 ± 2.8	0.30
VO2 at peak (ml/kg/min)	11.1 ± 3.9	10.7 ± 3.5	11.9 ± 4.8	0.46
VE/CO_2_slope	87 ± 69	96 ± 82	66 ± 18	0.29
% predicted peak VO_2_	36 ± 11	35 ± 11	36 ± 13	0.70
6 minute walk distance (m)	388 ± 108	405 ± 110	348 ± 96	0.19
Death during follow up (yr)	7 (23%)	4 (19%)	3 (33%)	0.13

RV = right ventricle. Values are expressed as mean ± SD or N (percent). *Non-normally distributed value expressed as median/interquartile range.

**Figure 1:**
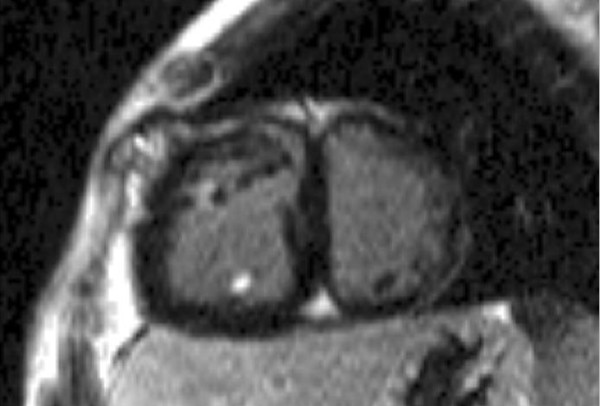
Examples of late gadolinium enhancement of a papillary muscle showing and discrete plaque in the right ventricular free wall and inferior interventricular junction.

**Figure 2:**
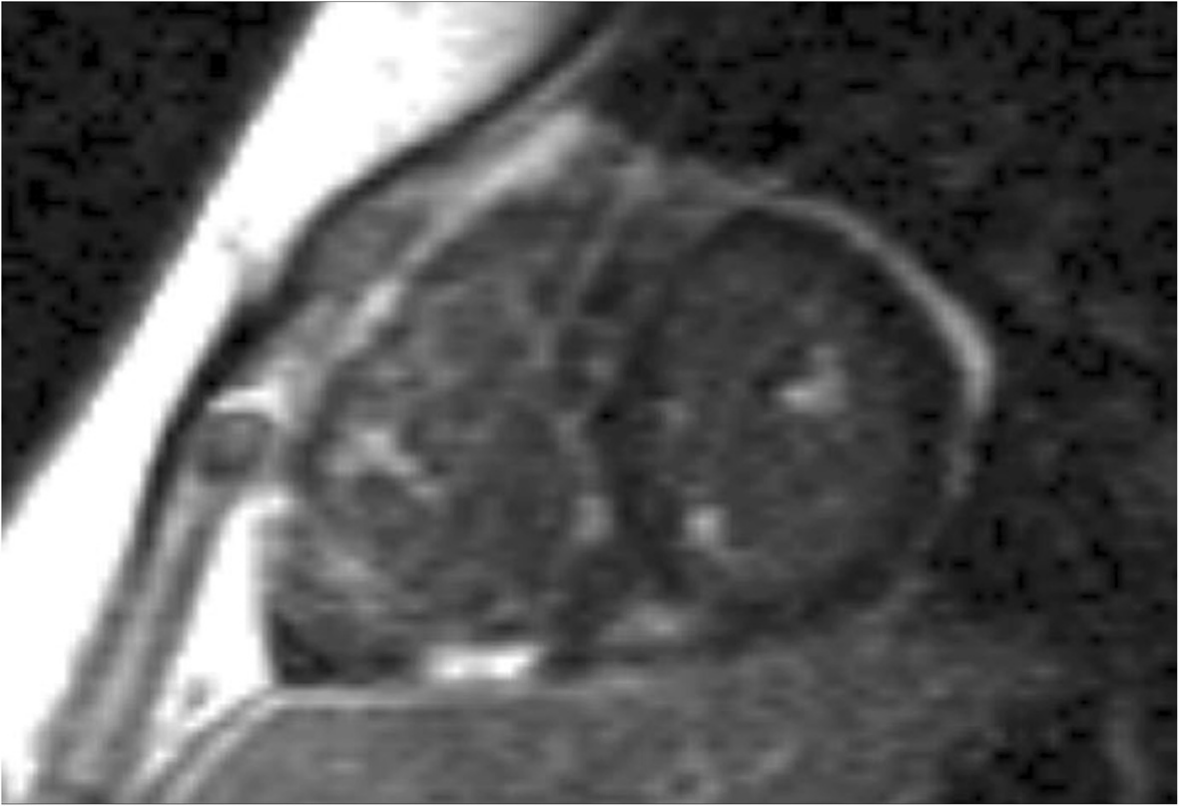
Late gadolinium enhancement in the right ventricular free wall as well as the RV-LV junction, which was not considered pathologic.

LGE findings were analyzed both qualitatively and quantitatively (Table 2). Qualitatively, any enhancement (other than RV-LV junction) was found in 22 (73%) patients. LGE was most prevalent in small areas of RV myocardium, found in 21 (70%), but also RV trabeculae in 18 (60%), LV myocardium in 10 (33%), and LV papillary muscles in 9 (30%) subjects. Examples are shown (Figure 1, Figure 2). All patients with LGE had enhancement of the RV wall except one, who had a small area of enhancement within the LV wall only. There were no typical patterns of enhancement (such as midwall fibrosis, probable surgical sites, or plausible coronary distribution). Only one patient had transmural enhancement in a small area of the inferior right ventricular free wall (Figure 2). Enhancement of the RV-LV junction was common (17 patients, 57%). Quantitatively, the median amount of LGE was 0.7% of myocardial volume (range 0.16 – 6.0%).

**Table 2:** Location and amounts of late gadolinium enhancement

	**Qualitative**			**Quantitative (% mass)**	
	**N**	**%**	**95% CI**	**Median**	**IQR**
Any myocardium*	22	73	56-86	0.72	1.62
LV myocardium	10	33	19-51	0.06	0.17
LV papillary	9	30	17-48	0.24	0.08
RV myocardium	21	70	52-83	0.31	0.76
RV trabeculae	18	60	42-75	0.33	1.11
RV-LV junction	17	56	39-73	0.86	0.70

*excluding RV-LV junction.

### Clinical significance of late enhancement

Qualitatively, those with any LGE did not differ in age, history of arrhythmia, oxygen saturation, nor hemoglobin, nor RV or LV size, mass, or function. Comparisons between those with and without RV enhancement are shown (Table 1), and similar comparisons for all other types of enhancement also showed no significant differences (data not shown). Exercise capacity (both maximal and submaximal) was also not different between patients with and without LGE. Quantitatively, no associations were found between quantity of LGE and other clinical variables (Table 3), including an analysis by location of enhancement.

**Table 3:** Correlations between baseline clinical, structural, and exercise metrics and the amount of late enhancement

**Correlation coefficients**	**Any myocardium**	**LV myocardium**	**LV papillary**	**RV myocardium**	**RV trabeculae**
**General characteristics**					
Age	0.04	0.22	−0.05	0.03	−0.08
NYHA class	−0.34	−0.29	−0.03	−0.36	−0.37
Heart rate at rest	−0.16	−0.10	−0.03	−0.17	−0.39
Oxygen saturation at rest	0.12	0.14	−0.01	0.12	0.22
Hematocrit	0.11	0.00	0.28	0.09	0.38
Hemoglobin	0.22	0.01	0.28	0.21	0.22
Brain natriuretic peptide	−0.12	−0.07	−0.22	−0.10	−0.12
**Cardiac morphology and function**					
RV end diastolic volume	0.02	−0.13	−0.21	0.08	0.23
RV end systolic volume	0.01	−0.16	−0.16	0.05	0.17
RV ejection fraction	0.06	0.09	0.07	0.05	0.01
RV mass	−0.05	−0.23	−0.01	−0.03	0.19
LV end diastolic volume	−0.04	0.09	−0.19	−0.02	−0.06
LV end systolic volume	−0.14	−0.05	−0.25	−0.12	−0.14
LV ejection fraction	0.26	0.24	0.30	0.23	0.19
LV mass	−0.04	0.03	−0.03	−0.04	−0.04
Pulmonary blood flow	−0.07	0.02	−0.08	−0.07	0.00
**Exercise capacity**					
Six minute walk distance	0.36	0.30	−0.01	0.38	0.33
Treadmill exercise duration	0.12	0.20	−0.06	0.13	0.00
Peak oxygen consumption	0.02	0.09	−0.14	0.04	−0.15
Percent predicted peak VO2	0.06	0.15	−0.07	0.06	−0.18
Ve/VCO2 slope	0.14	−0.06	0.20	0.13	0.01

RV = right ventricle, LV = left ventricle. None of the associations reached statistical significance.

### Ventricular size and function

**Figure 3:**
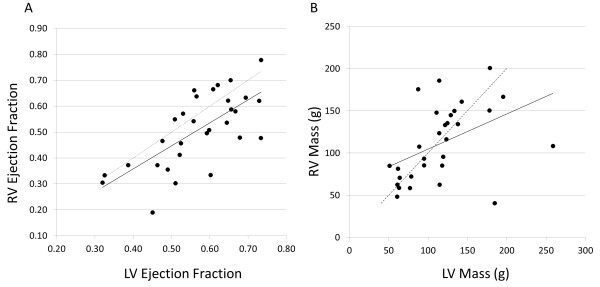
**Scatterplots showing the relationship between right and left ventricular ejection fraction (Panel A, left) and mass (Panel B, right).** Solid line is the linear trendline. Dotted line is the line of identity.

There was a correlation between RV and LV ejection fraction (r = 0.694, P < 0.001), with RV ejection fraction slightly lower than LV (Figure 3, left). All patients had severe RV hypertrophy (RV mass index 70 ± 26 g/m^2^) and RV mass correlated with LV mass (72 ± 27 g/m^2^, r = 0.448, P =0.0195, Figure 3, right). Two outliers were those with malposed great arteries.

### Survival

**Figure 4:**
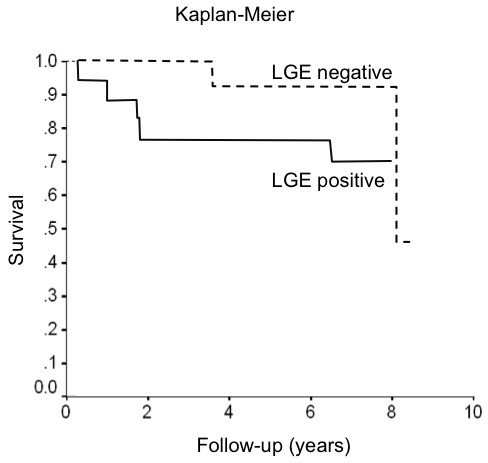
**Kaplan Meier curve showing survival in patients with versus without late gadolinium enhancement in the subendocardium.** There was no significant survival difference between the groups.

All patients were accounted for in follow-up (mean follow-up 7.4 ± 0.4 years for living patients). The longest duration of follow-up was 8.4 years. There were seven deaths total, five among patients with LGE present, two in patients without LGE findings. In addition there were three deaths in those who did not complete the LGE portion of the scan. Of those studied with LGE, three died of heart failure, but only one of these had detectable LGE. Two others died of non-cardiac causes (bladder carcinoma and acute abdomen). In two more the cause of death was unknown. The presence of LGE in any form did not differentiate survivors from non-survivors (log rank =0.03, P = 0.85). Kaplan Meier curves are shown (Figure 4). There were more early deaths in the LGE-positive group, but also more patients in this group, and no difference in survival was found even if events were censored after 4 years. Similarly, no significant differentiation was found based on location of enhancement.

## Discussion

The use of any imaging technique should be driven primarily by its ability to direct management decisions, inform disease mechanisms or estimate prognosis. Studies in congenital heart disease have demonstrated that LGE is associated with poorer functional class, ventricular volume and function, exercise capacity and arrhythmia [3-6,13]. In the present study of Eisenmenger patients, we found small amounts of fibrosis were very common, especially in the more vulnerable RV, indicating that mechanisms driving fibrosis are present. Quantified amounts were very small in part because of our decision to express values as a percentage of total RV and LV mass (given large VSDs in many patients). However, we could not find any meaningful clinical correlation with LGE, including clinical history, ventricular function, exercise capacity, and survival, despite multiple comparisons.

Identification of fibrosis demonstrates susceptibility to this final common pathway of ventricular dysfunction, particularly of the RV. Both chronic cyanosis and pressure loading likely contribute to various signaling pathways leading to myofibroblast activity that causes fibrosis [14-16]. Fibrosis in turn is generally associated with arrhythmia and myocardial dysfunction, which is a common cause of eventual death in ES as suggested by recent studies [1,17]. Diffuse fibrosis may be a potential target for pharmacotherapy, although LGE is unlikely to be sensitive enough to detect change. Many antifibrotic medications are vasodilators, which may be detrimental if they reduce systemic afterload more than pulmonary afterload and thereby promote right-to-left shunting.

There are several plausible explanations for the lack of association between LGE and clinical variables in this study. First, our sample was small. Although the study enrolled a limited number of patients, all data were acquired prospectively, and the cohort reflected the wide range of ES with long follow-up. While it is certainly possible that a larger sample size may show statistical differences, our data suggest that such differences may be small, even clinically insignificant. Despite the relatively smaller sample, we followed our patients for a considerably longer period of time compared to other studies, looking for clinical endpoints that may be related to the presence of fibrosis even in a small study. Yet after eight years of follow-up we could not show a meaningful association.

Second, while fibrosis is certainly present, it is likely more diffuse [8]. In performing these scans, it was challenging to appropriately null signal from normal myocardium. Diffuse non-replacement fibrosis affecting the bulk of the myocardium could create difficulties in identifying normal reference myocardium for nulling. In other studies, we have used T1 mapping methods to determine extracellular volume fraction and shown diffuse fibrosis to be present in 90% of in cyanotic congenital heart patients including ES. Importantly, the amount of fibrosis was often not detectable as dense replacement fibrosis using LGE in the same individuals [8]. Admittedly the quantitative methods used here were simplistic, and inaccuracies of measuring low amounts of fibrosis are possible. For diffuse fibrosis, LGE may not be the method of choice for detection and quantification in patients such as this [18].

Third, our methods of quantification may have been overly sensitive, suggested by our high prevalence of relatively small areas of fibrosis. Our initial aim was to be inclusive of any type of enhancement, not knowing whether clinical significance would be found. Images were acquired and interpreted by trained, experienced imagers, and we are reasonably confident in our ability to avoid artifacts, etc. Still, image quality can at times be limited, and interpretation was largely subjective. We specifically excluded enhancement due to blood pooling within trabeculae or RV-LV junctional enhancement due to hypertrophy [11], recognizing that contrast enhancement may not always implicate fibrosis. A contrary argument could be made that newer techniques with better sensitivity that have evolved since the start of this project could potentially show more abundant fibrosis.

Finally but perhaps most relevant to clinical practice, ES is a multi-organ disease, with many different causes of clinical symptoms, exercise capacity, and death. Fibrosis likely only plays a small role in the overall clinical trajectory of the patient. In fact, there were several non cardiac related deaths, and of the three patients with CHF-related death, two had no detectable LGE. Thus, it is possible that even in a larger population the presence of dense fibrosis in ES may still not have a demonstrable association between LGE and function or survival.

An additional novel finding from our study was that the RV and LV were morphologically and functionally similar, Mass and volume were linearly related; the LV had a proportionately larger mass, mostly due to inclusion of the septum. LGE was present in both ventricles but more abundant in the RV. The similarity of the two ventricles is congruous with the understanding that both face similar loading conditions from birth due to the shunt. The RV mass does not diminish in the postnatal period following a fall in pulmonary vascular resistance as occurs in those without shunts. Furthermore, the LGE data demonstrated fibrosis in both ventricles, not just the adversely loaded RV, as we have demonstrated in other conditions [4]. This is an important distinction from other forms of pulmonary arterial hypertension where the RV becomes disproportionately larger and more dysfunctional than the LV [18].

### Clinical relevance

Our study confirms the presence of myocardial fibrosis in ES, particularly in the morphologic RV, as found in smaller series [8]. Small amounts are consistent with the hypothesis that fibrosis is more diffuse in nature rather than dense replacement fibrosis. While this is important in considering the pathophysiology of ventricular dysfunction in this group, LGE does not appear to have significant clinical implications at present that would likely alter management. While laboratories may perform LGE routinely for every cardiac study, the data herein offer no support for this as routine practice in ES. Gadolinium-based contrast agents have an extensive history of relative safety, yet use has fallen under greater scrutiny in the years following the initiation of this study. While we know of no long-term adverse effect from Gadolinium administration in our patients, one cannot assume this would always be the case. Rare complications related to gadolinium administration may occur in the setting of renal insufficiency, for which the ES population is at risk [19]. There is also a remote chance of unintended systemic embolism from intravenous access in ES. These caveats should prompt hesitation before performing LGE routinely in this fragile population.

We note that CMR can offer valuable information for ES including clarification of anatomy, ventricular function, measurement of flow/shunts, and inspection of the pulmonary arteries including detection of thrombi [10]. However, prioritizing clinically relevant questions in the CMR protocol is important, including consideration of the relative value and potential risks of contrast administration.

## Conclusions

Although LGE is common in ES, it appears to be of limited extent, particularly in the RV. LGE did not correlate with functional capacity, severity of shunt or cyanosis, ventricular size or function, or arrhythmia in this study. Its routine application cannot be recommended at present by our pilot data. Further studies inclusive of measuring diffuse fibrosis in ES are warranted.

## Abbreviations

CMR: Cardiovascular magnetic resonance; ES: Eisenmenger syndrome; LGE: Late gadolinium enhancement; RV: Right ventricle; LV: Left ventricle.

## Competing interests

The authors declare that they have no competing interest.

## Authors’ contributions

CSB: Study design, funding, ethics, subject consent, study execution, CMR interpretation and quantification, data analysis and interpretation, and primary manuscript author. SKP: CMR acquisition and interpretation, data interpretation and manuscript review. CC: CMR quantification including quantification of late enhancement areas. SVB: Subject recruitment, CMR acquisition, CMR interpretation, manuscript review. KD: Follow up data for survival, manuscript review. MAG: Study design, hypothesis generation, funding, subject recruitment, data and manuscript review. All authors read and approved the final manuscript.
